# Spatial-temporal analysis of hospitalizations with death caused by oral cancer in Brazil and its correlation with the expansion of healthcare coverage

**DOI:** 10.4317/medoral.25470

**Published:** 2022-12-24

**Authors:** Hassan Lavalier de Oliveira Lima, Elisa Miranda Costa, Luciano de Andrade, Erika Barbara Abreu Fonseca Thomaz

**Affiliations:** 1 Orcid id:0000-0001-9394-4526. Graduate Program in Dentistry, Federal University of Maranhão, São Luís, MA, Brazil; 2 Orcid id:0000-0001-5364-0384. Postgraduate Program in Collective Health, Federal University of Maranhão, São Luís, MA, Brazil; 3 Orcid id:0000-0003-2077-1518. Department of Medicine, State University of Maringá, Paraná, PR, Brazil; 4 Orcid id:0000-0003-4156-4067. Graduate Program in Dentistry and Public Health, Federal University of Maranhão, São Luís, MA, Brazil

## Abstract

**Background:**

Oral cancer (OC) is a growing public health problem worldwide. In Brazil, the National Oral Health Policy, implemented in 2004, expanded access to oral health services and prioritized OC care. However, it is not known whether this expansion resulted in a reduction in hospital admissions with death. This study aimed to analyze the proportion of hospital admissions who progressed to death due to OC in Brazil from 2007 to 2019 and its correlation with the coverage of health services.

**Material and Methods:**

This study is an ecological, longitudinal, and analytical study of hospital admissions with death due to OC recorded in the Brazilian Hospital Information System. The following analyses were performed: descriptive, spatial (choropleth maps and Moran index), and negative binomial regression, with a hierarchical approach, estimating crude and adjusted regression coefficients (β) and respective 95% confidence intervals (95% CI) (alpha=5%).

**Results:**

In 2019, Moran's index (I) of spatial autocorrelation showed a negative association between hospital admissions with death and dentist surgeon/inhabitant rate (I=-0.176), physician/inhabitant rate (I=-0.157), family health strategy (FHS) coverage (I=-0.080), oral health team (OHT) coverage (I= -0.129), dental specialty centers (DSC)/inhabitant rate (I= -0.200), and oncology bed/inhabitant rate (I= -0.101). In the adjusted regression analysis, the proportion of hospitalizations with deaths caused by OC was higher in Brazilian states with a lower medical /inhabitant ratio (β= -0.014; *p*=0.040), a lower dentists/inhabitant ratio (β= -0.720; *p*=0.045), a lower number of DSC (β= -0.004; *p*<0.000), a lower amount paid per hospitalization (β= -10.350; *p*<0.001), and a lower number of biopsies (β= -0.00008; *p*=0.010). The proportion of hospitalizations that progressed to death showed a positive association with the number of days of hospitalization (β= 0.00002; *p*=0.002).

**Conclusions:**

Increased health care coverage has decreased serious hospital admissions with deaths caused by OC in Brazil.

** Key words:**Oral cancer, hospitalization, spatial analysis, death.

## Introduction

Oral cancer (OC) is a growing public health problem with a high morbidity and mortality rates (1). Inequalities in health care coverage hinder diagnosis and treatment, as well as worsen the prognosis of the disease, especially in lower-middle and upper-middle income countries, including most South American countries, such as Argentina, Bolivia, Colombia, Peru, and Brazil (2). This growth has been reflected in an increase in the number of outpatient treatments, in hospital admission rates, and in the public resources required to pay for the treatments (3).

In Brazil, the coverage of public oral health services has undergone a marked expansion since 2004, with the implementation of the National Oral Health Policy (NOHP) (4). This policy allowed for the implementation of prevention strategies, early diagnosis, and control of OC by means of the Family Health Strategy (FHS) and Oral Health Teams (OHT) as interventions of Primary Health Care (PHC) (5), as well as enabled the implementation of specialized dental care services throughout the country, through the creation of the Specialized Dental Centers (CEO, in Portuguese) in Oral Health (6).

Thus, the significant expansion of PHC in the poorest regions of the country sought to overcome social and geographic inequalities of access to health services, considering the principle of equity (7). However, despite the advances, especially in relation to the expansion of population coverage and the expansion of access to dental services, it is still possible to observe the presence of entry barriers in primary care, such as non-coverage of the Family Health Program, or the lack of doctors in basic health units and the long wait for secondary care, which reflects negatively on the morbidity and mortality indicators of OC (5).

Notably, the proportion of hospital deaths by OC can be an important indicator to evaluate healthcare systems, especially in relation to access to services, early diagnosis, integrality of actions, and resoluteness of suspected and confirmed cases of this neoplasm. It is in this context that research related to morbimortality caused by OC, in a spatial perspective, has been encouraged, mainly because it is considered an instrument for detecting failures in local health systems (8).

Thus, this study aimed to analyze the spatial-temporal correlation between the expansion of health service coverage and the proportion of hospital deaths caused by OC among Brazilian states and different regions in Brazil, identifying which elements of health service coverage relate to serious hospital admissions due to OC.

## Material and Methods

- Study design

This is an ecological, longitudinal, and analytical study, using secondary health data from 2007 to 2019, conducted in the 26 states and the Federal District of Brazil, with a focus on the spatial-temporal analysis of the data. The study population consisted of all records from hospitalizations that occurred in the Brazilian states, in which the main diagnosis noted in the hospital admission system was OC, which presented the ICD-10 code(s) (C00-C10) as the main diagnosis.

- Theoretical model

**Figure 1:**
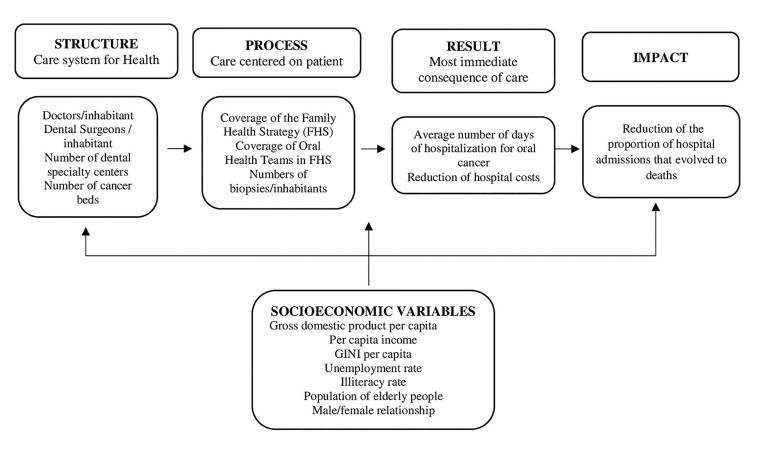
Theoretic model – variables presented in blocks: sociodemographic, structure of the facility, work process, results, and impact.

The magnitude and trend of hospitalization rates for OC are influenced by sociodemographic and socioeconomic aspects, in addition to the availability, efficacy, and quality of the treatment offered to patients (9). Structural elements, mainly comprised of human resources, physical infrastructure, and process elements, which reflect the daily practice of prevention and care delivery, suggest advances resulting from primary healthcare policies in reducing hospital admissions and expenditures, as well as with the improvement of living and health conditions among the Brazilian population (10). In the proposed model, the FHS and OHT coverage were considered indicators of the work process, since the FHS is a reorientation of the healthcare model. Therefore, it is assumed that the expansion of coverage contributes to the consolidation of the new healthcare delivery process. This theoretical model (Fig. 1) examines the relationship between structural elements, processes, and outcomes related to hospital admissions for OC, as well as the mediating effects of socioeconomic variables.

- Data Source

The data were categorized as indicators of sociodemographic, structural, and work process aspects (Table 1) related to OC. All data in the present study are of public domain and were used for the analysis of the completeness of hospital admissions and annual rates of expansion of health service coverage in primary, secondary, and tertiary care, together with spatial analysis and geoprocessing due to the complete availability of data in the Department of Informatics of the Unified Health System (DATASUS, in Portuguese).

- Data Analysis

This study used descriptive statistical methods, estimating measures of central tendency (percentages and means) and dispersion (95% confidence intervals - 95%CI - and standard deviations - SD). The spatial statistical analyses were developed in a Geographic Information System (GIS) environment. The data were collected in vector format, shapefile extension (shp), using the Universal Transverse Mercator Projection System (UTM) and Datum Geocentric Reference System for the Americas (SIRGAS), 2000.

Bivariate spatial autocorrelation analysis was used to demonstrate and explain the existing spatial association patterns concerning the distribution of the proportion of hospitalizations with deaths caused by OC among Brazilian regions and variables related to health service coverage. Spatial autocorrelation was performed using the Global Moran Index (I) (11).

For the regression analyses, the negative binomial hierarchical statistical model was used, due to the overdispersion of the data. The outcome was the proportion of deaths resulting from hospital admissions due to OC. This study used hierarchical modeling, according to the theoretical model described in Fig. 1, to enter the data into the model, with variables with *p*<0.10 remaining for subsequent blocks when the variables were entered into their respective block (12). The analyses were performed using the Stata 14.0 software (College Station, USA). For all analyses a significance level of 5% was adopted.

## Results

**Figure 2:**
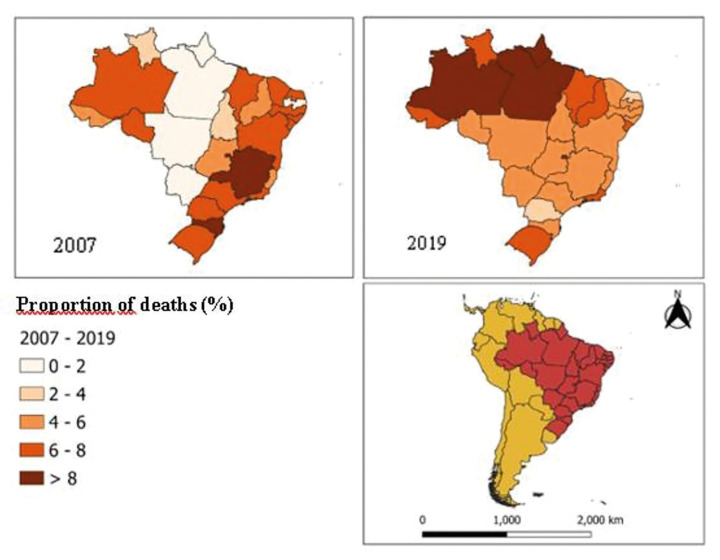
Proportion of hospital admissions for oral cancer (OC) that evolved to deaths in Brazil. 2007-2019.

**Figure 3:**
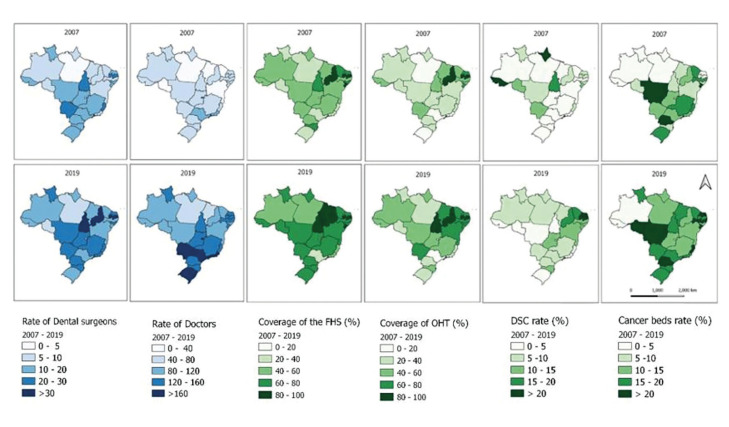
Healthcare coverage expansion: rate of dentists and doctors (100,000 inhabitants), of Family Health Strategy coverage (%), Oral Health Team coverage (%), rates of Dental Specialty Centers, and rate of oncology beds (100,000 inhabitants). Brazil. 2007-2019.

Data analysis showed that hospital admissions with death due to OC in Brazil were high and that there was a growing increase in hospital lethality due to the disease in the country. The states of the Northern region (Amazonas, Pará, and Amapá) presented the highest proportions of hospitalizations with deaths caused by OC, while the Northeastern region (Rio Grande do Norte and Piauí) presented the lowest proportions (Fig. 2). The spatial-temporal distribution of variables related to the expansion of health service coverage showed a significant increase in the number of dental surgeons and physicians per inhabitant registered in the Unified Health System (SUS, in Portuguese) between 2007 and 2018. The same was observed for FHS coverage and OHT coverage for all Brazilian states and the Federal District (Fig. 3).

Spatial autocorrelation indices in 2019 were negative for all analyzed predictors (Table 2). The regression analysis adjusted through hierarchical modeling (Table 3) showed that the proportion of hospitalizations with deaths was inversely associated with the ratio of physicians /inhabitant (β = -0.014; *p* = 0.040), ratio of dental surgeons /inhabitant (β = -0.720; *p* = 0.045), number of DSCs (β = -0.004; *p* < 0.001), average amount paid per hospital admission (β = -10.350; *p* < 0.001), and number of biopsies performed (β = -0.00008; *p* = 0.010). The proportion of hospitalizations with deaths showed a positive association with the number of days of hospitalization (β = 0.00002; *p* = 0.002) (Table 3).

**Table 1:**
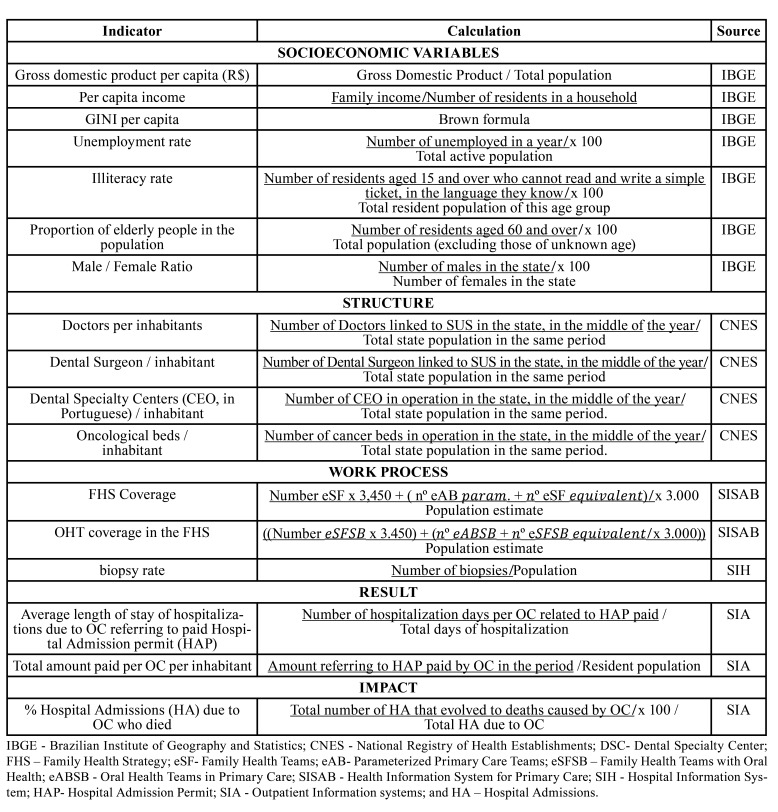
Indicators of the study. Brazil, by state, 2007 to 2019.

**Table 2:**
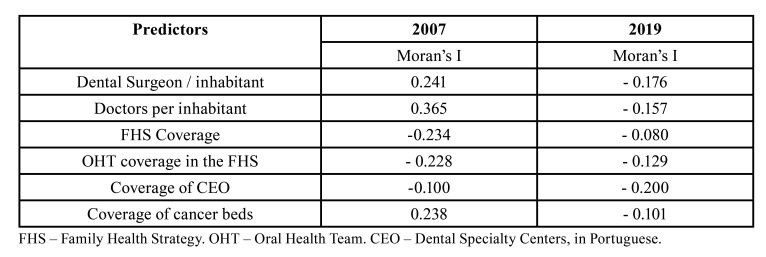
Global Moran Index (I) bivariate coefficient of the proportion of hospitalized patients per oral cancer according to variables related to the expansion of health service coverage. Brazil, 2007 and 2009.

**Table 3:**
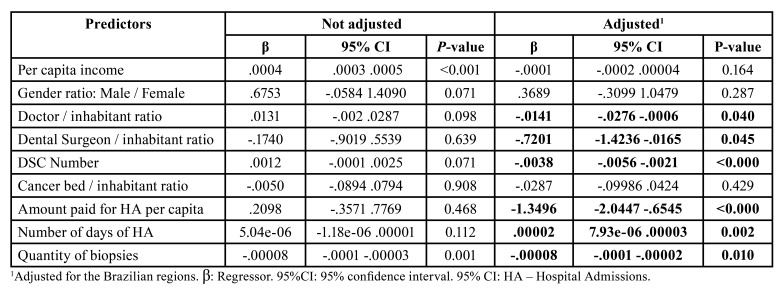
Association between health service coverage and the proportion of deaths among hospital admissions for oral cancer. Brazil, 2007-2019.

## Discussion

The results of the present study indicate a significant increase in registered cases of hospital admissions due to OC in Brazil between 2007 and 2019, with increasing hospital lethality for the disease in the country, presenting clear differences according to regions and Brazilian states. It is suggested that the observed increase in the number of hospital admissions may be related to the expansion of the health service network, which expanded oral health coverage in PHC through the implementation of CEO, encouraged by the NOHP (13).

The study of hospitalizations for OC is influenced by variations in the quantity and quality of health services provided to the population, in addition to factors related to the staging of the disease. Higher or lower levels of coverage, access, and effectiveness of health services can provide more favorable conditions for prevention, early diagnosis, and reduction of severe hospitalizations due to OC (14). This corroborates a study that highlighted the contribution of the NOHP in reduction of frequency of hospitalization for oral cancer (1). Investing in public oral health services may be an alternative to reduce social inequalities in health, and also provide dignity and access to prevention and treatment of OC.

The results of this study also showed that states with lower oral health coverage in PHC showed a higher number of hospitalizations and deaths caused by OC. In this study, the states in the Northern region of the country (Amazonas, Pará, and Amapá) had the highest proportions of hospitalizations and deaths from the disease. The Northern region concentrates the worst rates of the use of health services in the country and a low Human Development Index (HDI) in 46% of the health regions that make up the legal Amazon (15). Therefore, this increase in mortality caused by OC may be directly related to the shortage of health professionals, the advanced stage of the disease, and the lower availability of specialized oncology services in these regions.

More than 70% of OC cases occur in developing countries (16). Therefore, the measures adopted by the NOHP to prioritize regions with lower HDI may have a positive effect on the morbidity and mortality caused by OC in Brazil, which is a necessary alternative in order to reduce social inequalities in health, as well as provide dignity and access to OC prevention and treatment.

The Northeast region of the country presented the highest percentage of coverage of the FHS and OHT, and the lowest proportions of hospital deaths, highlighting the states of Rio Grande do Norte and Piauí. This runs in line with another study, which highlighted the contribution of NOHP in reducing the frequency of hospitalization due to OC (1). This result may have been influenced by changes in lifestyles with decreased exposure to risk factors or by an underreporting of deaths. Increased PHC coverage is associated with reduced mortality rates for OC and oropharynx (17). It is important to note that there was an increase in the number of OHTs after the implementation of the NOHP; however, the tertiary care network did not expand in the same manner (7).

The present study also showed that from 2007 to 2019 there was an expansion of oral health coverage in PHC and an increase in the number of CEO that may contribute to the increase in the resolvability rate of OC cases, thereby reducing the hospital demand for patients in advanced stages of lesions (4,5).

This study applied bivariate spatial autocorrelation methods to determine the spatial autocorrelation between the proportion of hospital admissions with deaths caused by OC and variables related to the health service coverage for 2007 and 2019. It is observed that in 2019 the spatial autocorrelation was negative or inverse for all analyzed predictors, that is, increasing the health service coverage at the three levels of care led to a lower hospital lethality caused by OC. Another Brazilian study found that higher rates of mortality due to OC were found in Brazilian states with lower FHS coverage and with a lower allocation of financial resources for actions aimed at PHC (13).

The significant expansion and maintenance of health service coverage over the last 12 years has led to an increase in the offer of a wide range of actions and services and has contributed to important positive effects on the health of the population (18), in addition to increasing the frequency of the early diagnosis of premalignant lesions (1).

In the adjusted binomial regression analysis, the states of the Northern region of Brazil, which are less developed, showed a higher probability of hospitalizations due to OC that evolves to death, illustrating the relationship of socioeconomic variables with this unfavorable outcome. Other studies have also identified socioeconomic and demographic indicators associated with mortality rates from the disease (19). However, this result may reflect less access to health services, or even less integration between levels of care, with negative effects on ensuring comprehensive, continuous, and good quality care to patients with this neoplasm. The obstacles to access to the public health network in a country like Brazil, where the less socioeconomically advantaged depend exclusively on these services, point to a direct relationship between the expansion of health service coverage and the reduction in hospitalization rates and mortality due to oral cancer (20).

Our findings highlighted that variables related to the ratio of dentists and physicians per inhabitants were associated with lower proportions of hospital admissions with deaths caused by OC. The supply of dental surgeons and physicians, who are the health professionals most qualified to perform the early diagnosis of OC, in basic health units increased from 2007 to 2019 (21).

The amount paid for hospital admissions has been associated with lower proportions of in-hospital deaths. Cancer treatment is expensive, and in recent decades the cost has skyrocketed, especially in developed countries, partly as a result of new investments in new diagnostic and therapeutic approaches (2).

The greater coverage of family health teams, OHT in the family health strategy, CEO, and oncology beds, as well as a greater performance of biopsies, were associated with lower proportions of hospitalizations with deaths caused by OC. These results show the need to maintain investments in the area in order to change the prognosis of this disease, which so severely affects the population. A health service focused on health promotion, disease prevention, and early diagnosis, performed by an interdisciplinary team, can provide a reduction in the lethality and mortality caused by OC (22).

The main limitation of the present study was the use of secondary data, aggregated to the level of the Brazilian states and the Federal District. Secondary data obtained from DATASUS have limitations regarding the adequate registration of cases and treatments. Incorrect filling out and filing of the system’s records, or filling out the records after the deadline stipulated by the Ministry of Health, may result in inconsistencies.

To the best of our knowledge, this is the first nationwide study with a spatial approach that sought to identify the relationship between health service coverage and hospital lethality caused by OC in order to analyze whether the expansion of access and health service coverage would have effects on the reduction of potentially severe cases (that evolved to death). The study included hundreds of thousands of cases, and the databases were organized month by month, allowing for a more accurate monitoring of the associations.

The results of this study have important implications for the healthcare model in Brazil and in other countries, especially those that seek to base their national health systems more strongly on PHC. The present study suggests that expanding and consolidating health service coverage can increase OC patients’ access to comprehensive care, in turn reducing morbidity and mortality, and consequently, the number of days of hospitalization and health system costs. Based on the proper knowledge of the epidemiological profile, what is needed is an intensification of public policies aimed at this pathology.
